# Kernel machine SNP set analysis finds the association of BUD13, ZPR1, and APOA5 variants with metabolic syndrome in Tehran Cardio-metabolic Genetics Study

**DOI:** 10.1038/s41598-021-89509-5

**Published:** 2021-05-13

**Authors:** Sajedeh Masjoudi, Bahareh Sedaghati-khayat, Niloufar Javanrouh Givi, Leila Najd Hassan Bonab, Fereidoun Azizi, Maryam S. Daneshpour

**Affiliations:** 1 Cellular and Molecular Endocrine Research Center, Research Institute for Endocrine Sciences, Shahid Beheshti University of Medical Sciences, PO Box 19195-4763, Tehran, Iran; 2 Endocrine Research Center, Research Institute for Endocrine Sciences, Shahid Beheshti University of Medical Sciences, Tehran, Iran

**Keywords:** Genetics, Molecular medicine

## Abstract

Metabolic syndrome (MetS) is one of the most important risk factors for cardiovascular disease. The 11p23.3 chromosomal region plays a potential role in the pathogenesis of MetS. The present study aimed to assess the association between 18 single nucleotide polymorphisms (SNPs) located at the *BUD13, ZPR1*, and *APOA5* genes with MetS in the Tehran Cardio-metabolic Genetics Study (TCGS). In 5421 MetS affected and non-affected participants, we analyzed the data using two models. The first model (MetS model) examined SNPs' association with MetS. The second model (HTg-MetS Model) examined the association of SNPs with MetS affection participants who had a high plasma triglyceride (TG). The four-gamete rules were used to make SNP sets from correlated nearby SNPs. The kernel machine regression models and single SNP regression evaluated the association between SNP sets and MetS. The kernel machine results showed two sets over three sets of correlated SNPs have a significant joint effect on both models (p < 0.0001). Also, single SNP regression results showed that the odds ratios (ORs) for both models are almost similar; however, the p-values had slightly higher significance levels in the HTg-MetS model. The strongest ORs in the HTg-MetS model belonged to the G allele in rs2266788 (MetS: OR = 1.3, p = 3.6 × 10^–7^; HTg-MetS: OR = 1.4, p = 2.3 × 10^–11^) and the T allele in rs651821 (MetS: OR = 1.3, p = 2.8 × 10^–7^; HTg-MetS: OR = 1.4, p = 3.6 × 10^–11^). In the present study, the kernel machine regression models could help assess the association between the BUD13, ZPR1, and APOA5 gene variants (11p23.3 region) with lipid-related traits in MetS and MetS affected with high TG.

## Introduction

Metabolic syndrome (MetS) is a combination of central obesity, hypertension, dyslipidemia, and insulin resistance which could increase the risk of type two diabetes (T2D) fivefold and is associated with a doubled risk of developing cardiovascular disease (CVD)^1–4^. It has been debated whether the combination of metabolic characteristics referred to as MetS represents a biologically meaningful entity in itself^5,6^ and generally accepted that the current criteria of MetS are integrated assessment strategies with only a binary outcome rather than precise levels of metabolic components^7^.

Many factors are involved in pathophysiological mechanisms of Mets, such as ectopic fat deposition, excess adipose tissue mass, the excessive flux of fatty acids, and inflammation.

As hyperlipidemia is the most frequent Mets component, some critical effects were reported on its pathophysiological mechanisms such as ectopic fat deposition, excess adipose tissue mass, the excessive flux of fatty acids, and inflammation^3,8^.

Apolipoproteins play a crucial role in blood, cerebrospinal fluid, and lymph lipid transportation.

In lipid transportation in the blood and cerebrospinal fluid and lymph, apolipoproteins play an essential role.

The previous genome-wide association analysis showed a genomic locus in correlation with MetS and its components in 11q23.3^9^. This region encodes genes involved in lipid metabolism, and previous studies indicated that several single nucleotide polymorphisms (SNPs) located in *ZPR1* Zinc Finger (Gene ID: 8882; OMIM: 603901), *BUD13 homolog* (Gene ID: 84811), and *APOA5* (Gene ID: 116519; OMIM: 606368) have a significant association with quantitative phenotypes such as lipid profile components^10–13^.

*ZPR1* is an essential regulatory protein that is responsible for normal cell proliferation and signal transduction and acts as a signaling molecule transporting proliferative growth signals from the cytoplasm to the nucleus. For normal nuclear function in cell proliferation and signal transduction, *ZPR1* is an essential regulatory protein that acts as a signaling molecule that communicates proliferative growth signals from the cytoplasm to the nucleus^14^.

Peroxisome proliferator-activated receptor gamma (*PPARG*) and the hepatocyte nuclear factor 4 alpha (HNF4a, nuclear receptor 2A1) bind to *ZPR1* promoter activation of various visceral systems genes in, involving in glucose, fatty acid, and cholesterol metabolism^15,16^. *BUD13* was identified in yeast as a splicing factor that affects nuclear pre-mRNA retention; however, nowadays, this region is known as a member of the RES complex^17,18^ and its functionality is controversial. The increase of plasma triglyceride (TG) levels could be a major risk factor for coronary artery disease, and *APOA5* is an essential determinant of its level. The *APOA5* gene is the closest member of the APO gene cluster to the *BUD13/ZPR1* gene cluster, which its variations are well known as the SNPs associated with an increased risk of coronary artery disease metabolic syndrome^19–21^.

The genetic variants of the *BUD13, ZPR1*, and *APOA5* genes concerning cardio-metabolic phenotypes were reported in different populations^10,11^, but only a few studies have been conducted in the Middle Eastern populations. Therefore, we performed this study to estimate the association of the *BUD13, ZPR1*, and *APOA5* genetic variations on the Tehran Cardio-metabolic Genetics Study (TCGS) population^21^. This study focuses on replicating the genetic markers associated with metabolic syndrome and determining their effects on triglyceride concentration among MetS affected subjects in an Iranian population^22^.

## Results

In the first model of analysis (MetS versus non-Mets) 2996 individuals were affected with MetS, and 2425 individuals were non-affected. The MetS group was older with higher mean values for MetS components except for HDL concentration. About 0.7% and 35% were Lipid-lowering drug users, 3.5% and 35% were hypertension-lowering drug users, and 1.7% and 25% were diabetes drug users among MetS and non-MetS participants, respectively (Table 1).

**Table 1:** Summary of demographic and clinical characteristics of study individuals.

	Unrelated individuals (n = 5421)					
	Non MetS (n = 2425)			MetS (n = 2996)		
	TG < 150 (n = 2170)^a^	TG ≥ 150 (n = 255)^b^	All (n = 2425)^c^	TG < 150 (n = 453)^d^	TG ≥ 150 (n = 2543)^e^	All (n = 2996)^f^
Age (years)	41.2 ± 15.1	42.2 ± 12.4	41.3 ± 14.9	56.2 ± 14.7	55.7 ± 14.4	55.85 ± 14.4
Male (%)	857 (39.4)	145 (56.8)	1002 (41.3)	215 (47.4)	1225 (48.1)	1440 (48)
Fast blood sugar (mg/dl)	90.6 ± 14.6	89.37.5	90.5 ± 14.1	108.6 ± 29.3	112.6 ± 39	112 ± 37.7
Waist circumference (cm)	89.8 ± 11.5	89.9 ± 9.9	89.8 ± 11.3	102.3 ± 9.5	101.2 ± 10.2	101.3 ± 10.1
Hip circumference (cm)	99.9 ± 9.1	98.1 ± 8	99.7 ± 9	104.7 ± 10.2	103.2 ± 9.3	103.4 ± 9.5
Body Mass Index	26.4 ± 4.6	26.4 ± 3.8	26.4 ± 4.6	30.8 ± 4.8	30.2 ± 4.7	30.3 ± 4.7
Diastolic blood pressure	73.3 ± 9.1	73.6 ± 7.7	73.3 ± 8.9	81.9 ± 10.9	79.4 ± 10.1	79.8 ± 10.2
Systolic blood pressure	108.6 ± 14.6	108.5 ± 10.3	108.6 ± 14.2	129.1 ± 17.6	123.7 ± 17.1	124.5 ± 17.3
Triglyceride (mg/dl)	93.2 ± 28.3	194.3 ± 55	103.8 ± 44.7	111.3 ± 24.2	198.8 ± 116	185.5 ± 111.7
HDL-C (mg/dl)	51 ± 10.4	45.7 ± 9.9	50.5 ± 10.5	43.85 ± 9.4	42.2 ± 10.6	42.5 ± 10.4
Cholesterol (mg/dl)	178.4 ± 34.4	205 ± 36.6	181.2 ± 35.6	180.8 ± 36.7	188.7 ± 44	187.5 ± 43.1
LDL-C (mg/dl)	105.2 ± 27.7	123.9 ± 30.4	107.2 ± 28.6	112 ± 29.6	111.6 ± 35.5	111.7 ± 34.6
non-HDL-C (mg/dl)	127.3 ± 32.3	159.3 ± 35.4	130.7 ± 34.1	136.9 ± 33.9	146.3 ± 43.7	144.9 ± 42.5
Lipid lowering drug user (%)	0	16 (6.2)	16 (0.7)	0	1048 (41.2)	1048 (35)
Hypertension lowering drug user (%)	86 (4)	0	86 (3.5)	139 (30.6)	898 (35.3)	1037 (34.7)
Diabetes drug user (%)	38 (1.8)	4 (1.6)	42 (1.7)	82 (18.1)	629 (24.7)	711 (23.8)

Values expressed as mean ± SD or number (%).

*LDL-C* low-density lipoprotein cholesterol, *HDL-C* high-density lipoprotein cholesterol.

p-values of t-student or chi-square test between the case and control groups in both models were significant (p < 0.001).

^a^HTg-MetS model control group.

^b^Excluded from HTg-MetS model.

^c^MetS model control group.

^d^Excluded from HTg-MetS model.

^e^HTg-MetS model control group.

^f^MetS model case group.

In the HTg-MetS model, comparing the 2543 cases and 2170 controls showed a significant difference within all metabolic syndrome risk factors. In this case, all considered risk factors for MetS had higher mean values in the case group. Between lipid drug users in the case and control groups, a significant difference is observed. None of the case group subjects used lipid-lowering drugs, but 41.2% of controls that consumed drugs indicated that the lipid-lowering drug consumption is directly related to the increased TG levels in HTg-MetS individuals. Table 1 summarizes the demographic and clinical characteristics of the studied individuals for both analytical models.

**Figure 1:**
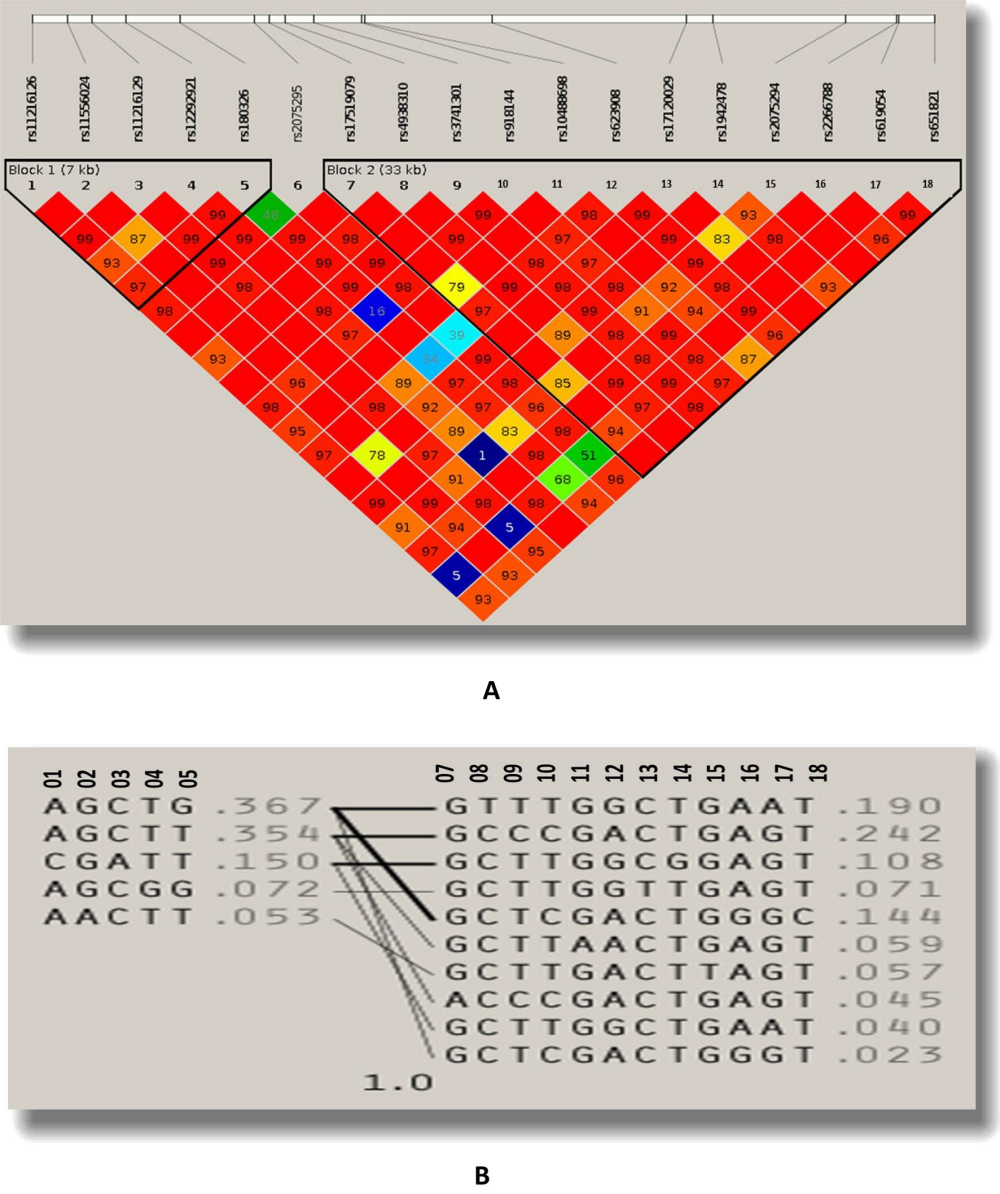
Linkage Disequilibrium blocks of 18 tag SNPs of *BUD13/ZPR1/APOA5* in the chromosomal region (11q23.3) (3.3 Kb) according to four gamete rules, LD plot of Haploview software 2(A) and Haplotypes 2(B).

**Figure 2:**
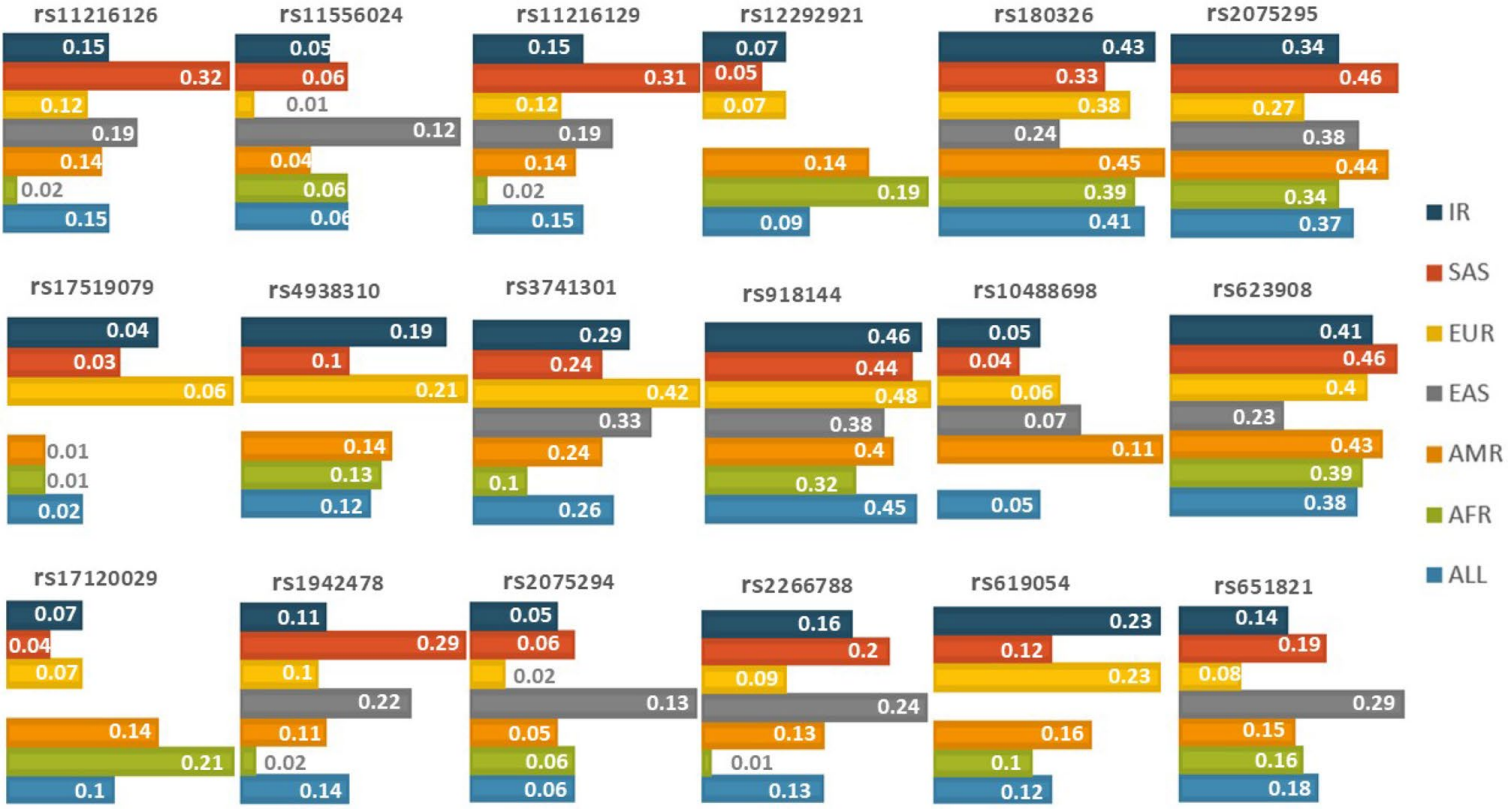
Compares MAF (Minor allele frequency) for 18 SNPs for different populations, Iranian population (IR), and Global minor allele frequency (ALL).

The selected SNPs were clustered into three variant sets according to the four gamete rules procedure with the sizes of 5, 1, and 12, shown in Table 2. Figure 1 presents the LD and haplotype plots related to the four gamete rules procedure. Moreover, Fig. 2 compares 18 selected SNPs' allele frequencies among populations from five continents with our population.

**Table 2:** Characteristics of the Markers in selected chromosomal region.

Gene	Consequence	SNP	Set name	Position	Major:minor	MAF^a^	GMAF^b^	Risk allel	RAF^C^
BUD13	Downstream gene variant	rs11216126	S1	11:116746524	A:C	0.15	0.15	C	0.15
	3 Prime UTR variant	rs11556024		11:116748335	G:A	0.05	0.06	A	0.05
	Intronic variant	rs11216129		11:116749540	C:A	0.15	0.15	A	0.15
	Intronic variant	rs12292921		11:116751247	T:G	0.07	0.09	G	0.07
	Intronic variant	rs180326		11:116753987	T:G	0.43	0.41	G	0.43
	Intronic variant	rs2075295	S2	11:116757685	T:C	0.34	0.37	C	0.34
	Intronic variant	rs17519079	S3	11:116758437	G:A	0.04	0.02	A	0.04
	Intronic variant	rs4938310		11:116759233	C:T	0.19	0.12	C	0.81
	Intronic variant	rs3741301		11:116760675	T:C	0.29	0.001	T	0.71
	Synonymous variant	rs918144		11:116763109	T:C	0.46	0.45	C	0.46
	Missense variant	rs10488698		11:116763231	G:A	0.05	0.05	A	0.05
	Downstream gene variant	rs623908		11:116769652	A:G	0.41	0.4	A	0.59
ZPR1	Intronic variant	rs17120029		11:116779402	C:T	0.07	0.1	T	0.07
	Intronic variant	rs1942478		11:116780747	T:G	0.11	0.1	T	0.89
	Intronic variant	rs2075294		11:116787406	G:T	0.05	0.06	T	0.05
ZPR1/APOA5	ZPR1: 2 KB Upstream Variant APOA5 : 3 Prime UTR Variant	rs2266788		11:116789970	A:G	0.16	0.13	G	0.16
	ZPR1: 2 KB Upstream Variant APOA5 : 3 Prime UTR Variant	rs619054		11:116790097	G:A	0.23	0.12	G	0.77
APOA5	5 prime UTR variant	rs651821		11:116791863	T:C	0.14	0.18	C	0.14

^a^Minor allele frequency in Iranian population.

^b^Global minor allele frequency.

^c^Risk allele frequency.

The kernel machine regression between SNP sets and case–control groups in the MetS model showed that the first and the last sets had a significant association with MetS (p-value < 0.0001). This finding were confirmed in the HTg-MetS model with the same significance level. In both models, most SNPs showed the association, especially two variations of the APOA5 gene: rs2266788, MetS model: [OR = 1.3, p = 3.66E − 07], HTg-MetS model: [OR = 1.4, p = 2.32E − 11] and rs651821, MetS model: [OR = 1.3, p = 2.86E − 07], HTg-MetS model: [OR: 1.4, p = 3.63E − 11] (Tables 3 and 4).

**Table 3:** Association result for MetS model.

Set name	SNP	LKMR^a^ p-value	Beta	SE	CI^c^ (lower, upper)		OR ^b^	Max (T)^d^ p-value
S1	rs11216126	< 0.0001	− 0.062	0.05284	− 0.1659	0.04124	0.9	0.2382
	rs11556024		− 0.067	0.0846	− 0.2329	0.09873	0.9	0.4278
	rs11216129		− 0.057	0.05328	− 0.1616	0.04726	0.9	0.2833
	rs12292921		0.2862	0.07402	0.1411	0.4313	1.3	0.0001
	rs180326		0.1293	0.0387	0.05347	0.2052	1.1	0.0008
S2	rs2075295	0.938	− 0.003	0.0399	− 0.08132	0.07511	0.9	0.93
S3	rs17519079	< 0.0001	− 0.057	0.0894	− 0.2319	0.1185	0.9	0.52
	rs4938310		− 0.133	0.04784	− 0.2263	− 0.03876	0.8	0.005
	rs3741301		− 0.056	0.04173	− 0.1374	0.0262	0.9	0.18
	rs918144		0.0974	0.0381	0.0227	0.1721	1.1	0.01
	rs10488698		− 0.119	0.07948	− 0.2751	0.0365	0.8	0.13
	rs623908		− 0.042	0.03834	− 0.1176	0.03275	0.9	0.26
	rs17120029		0.245	0.07447	0.09909	0.391	1.2	0.0009
	rs1942478		− 0.105	0.05997	− 0.2225	0.01254	0.9	0.07
	rs2075294		− 0.098	0.08	− 0.255	0.05858	0.9	0.21
	rs2266788		0.2609	0.05131	0.1604	0.3615	1.3	3.663E − 07
	rs619054		− 0.097	0.04493	− 0.1854	− 0.009276	0.9	0.03
	rs651821		0.2773	0.05403	0.1714	0.3832	1.3	2.867E − 07

^a^Logistic Kernel machine regression.

^b^Odd ratio for minor allele.

^c^95% Confidence interval for BETA.

^d^MAX(T) p-value with 1,000,000 replications.

**Table 4:** Association result for HTg-MetS model.

Set name	SNP	LKMR^a^ p-value	Beta	SE	CI^c^ (lower, upper)		OR^b^	Max (T)^d^ p-value
S1	rs11216126	< 0.0001	− 0.1095	0.05718	− 0.2216	0.002587	0.8	0.05553
	rs11556024		− 0.05451	0.0909	− 0.2327	0.1236	0.9	0.5487
	rs11216129		− 0.1049	0.05772	− 0.2181	0.008186	0.9	0.06904
	rs12292921		0.331	0.07953	0.1751	0.4869	1.3	0.00003
	rs180326		0.1724	0.04191	0.09029	0.2546	1.1	0.00003
S2	rs2075295	0.944	− 0.003	0.04306	− 0.08741	0.0814	0.9	0.94
S3	rs17519079	< 0.0001	− 0.1562	0.09806	− 0.3484	0.03601	0.8	0.11
	rs4938310		− 0.1845	0.05172	− 0.2858	− 0.08309	0.8	0.0003
	rs3741301		− 0.09505	0.04517	− 0.1836	− 0.00651	0.9	0.03
	rs918144		0.13	0.04119	0.04925	0.2107	1.1	0.001
	rs10488698		− 0.07839	0.0851	− 0.2452	0.08839	0.9	0.35
	rs623908		− 0.08625	0.04155	− 0.1677	− 0.00481	0.9	0.03
	rs17120029		0.299	0.08015	0.1419	0.4561	1.3	0.0001
	rs1942478		− 0.1816	0.06504	− 0.3091	− 0.05415	0.9	0.005
	rs2075294		− 0.08536	0.08589	− 0.2537	0.08298	0.9	0.32
	rs2266788		0.3747	0.05605	0.2648	0.4845	1.4	2.32E − 11
	rs619054		− 0.1348	0.04835	− 0.2296	− 0.04005	0.8	0.005
	rs651821		0.3901	0.05894	0.2746	0.5056	1.4	3.63E − 11

^a^Logistic Kernel machine regression.

^b^Odd ratio for minor allele.

^c^95% confidence interval for BETA.

^d^MAX(T) p-value with 1,000,000 replications.

Four SNPs (rs2266788, rs651821, rs12292921, and rs180326) with the highest significance levels were selected to indicate the relation between MetS components level variation and the number of risk alleles. The result of association analysis showed the significant difference between studied markers and the triglyceride and HDL-C level (p < 0.001), and three of them were in association with non-HDL-C levels (p < 0.001) (Table 5).

**Table 5:** Single SNPs association analyses for metabolic components (mean ± SD).

	rs12292921			rs180326			rs2266788			rs651821		
	TT	GT	GG	TT	GT	GG	AA	GA	GG	TT	CT	CC
Fast blood sugar (mg/dl)	102 ± 31.5	102.7 ± 29.8	97.8 ± 14.9	101.7 ± 30.4	102.3 ± 32	101.8 ± 30.4	102.2 ± 31	101.5 ± 30.3	104.5 ± 40.7	102.1 ± 31	101.6 ± 31.4	104.8 ± 35.5
Waist circumference (cm)	95.5 ± 12.6	95.8 ± 12.5	99.6 ± 13.7	95.6 ± 12.3	95.6 ± 12.9	95.4 ± 12.5	95.7 ± 12.5	95.1 ± 12.9	95.4 ± 12.3	95.7 ± 12.6	95 ± 12.8	96.7 ± 12.9
Diastolic blood pressure	76.6 ± 10.3	77.3 ± 10.9	76.9 ± 10.4	76.4 ± 10.2	76.7 ± 10.6	76.8 ± 10.2	76.8 ± 10.4	76.2 ± 10.4	76.7 ± 10	76.8 ± 10.4	76.2 ± 10.6	77.3 ± 9.7
Systolic blood pressure	116.9 ± 18	117.9 ± 17.8	114.5 ± 15.6	116.9 ± 17.7	117.3 ± 18.2	116.5 ± 17.5	117.1 ± 17.9	116.7 ± 18.1	116.3 ± 16.8	117 ± 17.9	116.8 ± 18.1	117.6 ± 17.4
Cholesterol (mg/dl)	184.5 ± 39.9	183.2 ± 40.5	186 ± 41.2	184.5 ± 40.4	184.2 ± 39.4	184.6 ± 40	183.5 ± 39.8	186.1 ± 39.7	186.5 ± 44.6	183.6 ± 39.8	186.2 ± 40.3	186.2 ± 38.5
Triglyceride (mg/dl)	**146.3 ± 98.2**	**153.6 ± 82.2**	**197.8 ± 124.3**	**136 ± 69.1**	**150 ± 90.2**	**160.6 ± 139.9**	**138.9 ± 76**	**162.1 ± 99.2**	**215 ± 277.3**	**140.1 ± 77.8**	**163.8 ± 128.8**	**205.5 ± 158.4**
HDL-C (mg/dl)	**46.4 ± 11.2**	**45.2 ± 11.1**	**42.1 ± 14.3**	**47.3 ± 10.9**	**46 ± 11.3**	**44.9 ± 11.4**	**46.8 ± 11.2**	**45.1 ± 11.2**	**42 ± 10.8**	**46.8 ± 11.2**	**45 ± 11.3**	**41.2 ± 10.6**
LDL-C (mg/dl)	109.6 ± 32	108.6 ± 32.5	107 ± 35.4	109.8 ± 32.8	109.2 ± 31.7	109.3 ± 31.9	109 ± 32.3	110.6 ± 31.9	108.2 ± 30.2	109 ± 32.3	110.5 ± 32	110 ± 27.6
Non-HDL-C (mg/dl)	138 ± 39.6	137.9 ± 39.5	143.8 ± 39.8	**137.2 ± 39.8**	**138.1 ± 39.2**	**139.3 ± 39.5**	**136.7 ± 39.1**	**140.9 ± 40.3**	**142.8 ± 39.2**	**136.8 ± 39.3**	**140.9 ± 40**	**145 ± 39.3**

Bold values: p-values of between genotype groups analysis were significant (p < 0.001).

**Figure 3:**
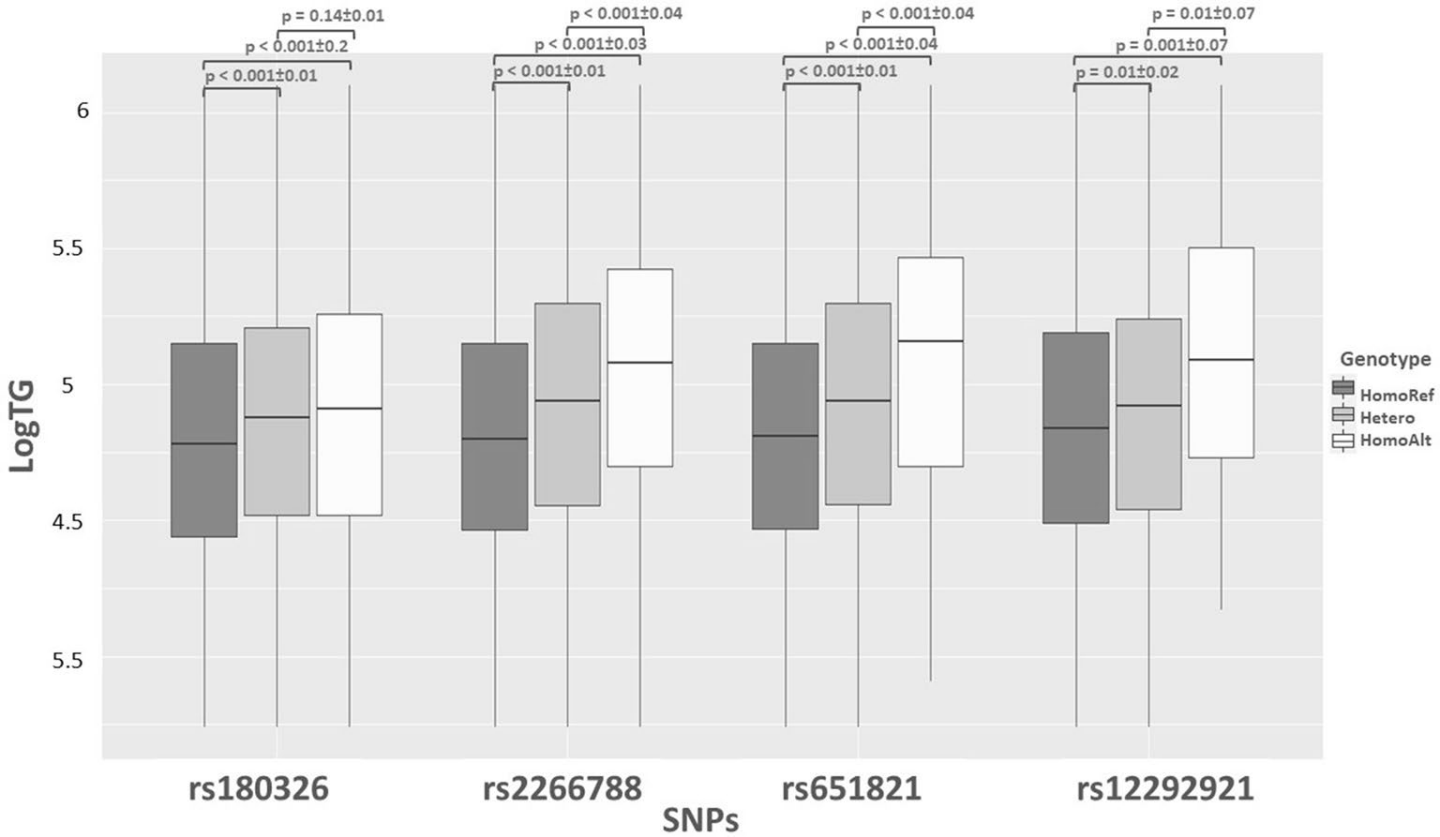
Log of TG levels (LogTG) against the four notable significant SNPs. This figure also shows TG levels by increasing risk alleles in each genotype type in subjects. The p-values and standard errors for between genotypes can be found (p-value ± S.E).

Figure 3 tries to present that the TG has the highest level in the HomoAlt groups for all four SNPs, and there is an association between raised TG levels in MetS-affected and the presence of the risk allele. The SNPs were tested for linkage disequilibrium (LD) to indicate the same signal between rs2266788 and rs651821 in the S3 and showed a strong LD (D′ > 0.9; r^2^ > 0.8).

## Discussion

This is the first kernel machine study to evaluate association genetic variations within the *BUD13, ZPR1*, and *APOA5* genes (11q23.3 region) with MetS and HTg-MetS. This kernel machine is a logistic sequence kernel association test that is denoted as SKAT method. SKAT is a supervised, flexible, computationally efficient regression method that handles tests for evaluating the association between common and rare genetic variants within a region and a continuous or dichotomous trait while easily adjusting for covariates. This method is also useful for assessing the association between joint effects of all SNPs within a set and the considered phenotype. Moreover, SKAT can quickly calculate calibrated p-values for rare variant association analysis in case–control studies. This regression-based method overcomes the limited power of classical single-marker association analysis for rare variants poses a central challenge in association studies^23,24^.

The results of both the kernel machine and MAX (T) tests in our study show that rs2266788, which belongs to both ZPR1 and APOA5 genes, is associated with MetS and MetS affected subjects that had a high TG. Furthermore, MetS affected subjects with homozygote G allele in rs2266788 have 1.4 times high risk for Tg increasing. These findings are consistent with similar studies on Korean and Kuwaiti populations^25,26^.

For the studied markers, most of the Iranian allele frequencies were common in the population, and the association result confirms the result of previous studies^7,13,25,26^. According to the association models, the S1 and S3 sets demonstrated a significant correlation with MetS and the high TG model. The two lowest p-values belong to rs2266788 (*ZPR1*/*APOA5* genes) and rs651821 (*APOA5* gene), to increase the risk of High TG 1.4 times in the case group of the HTg-MetS model.

**Figure 4:**
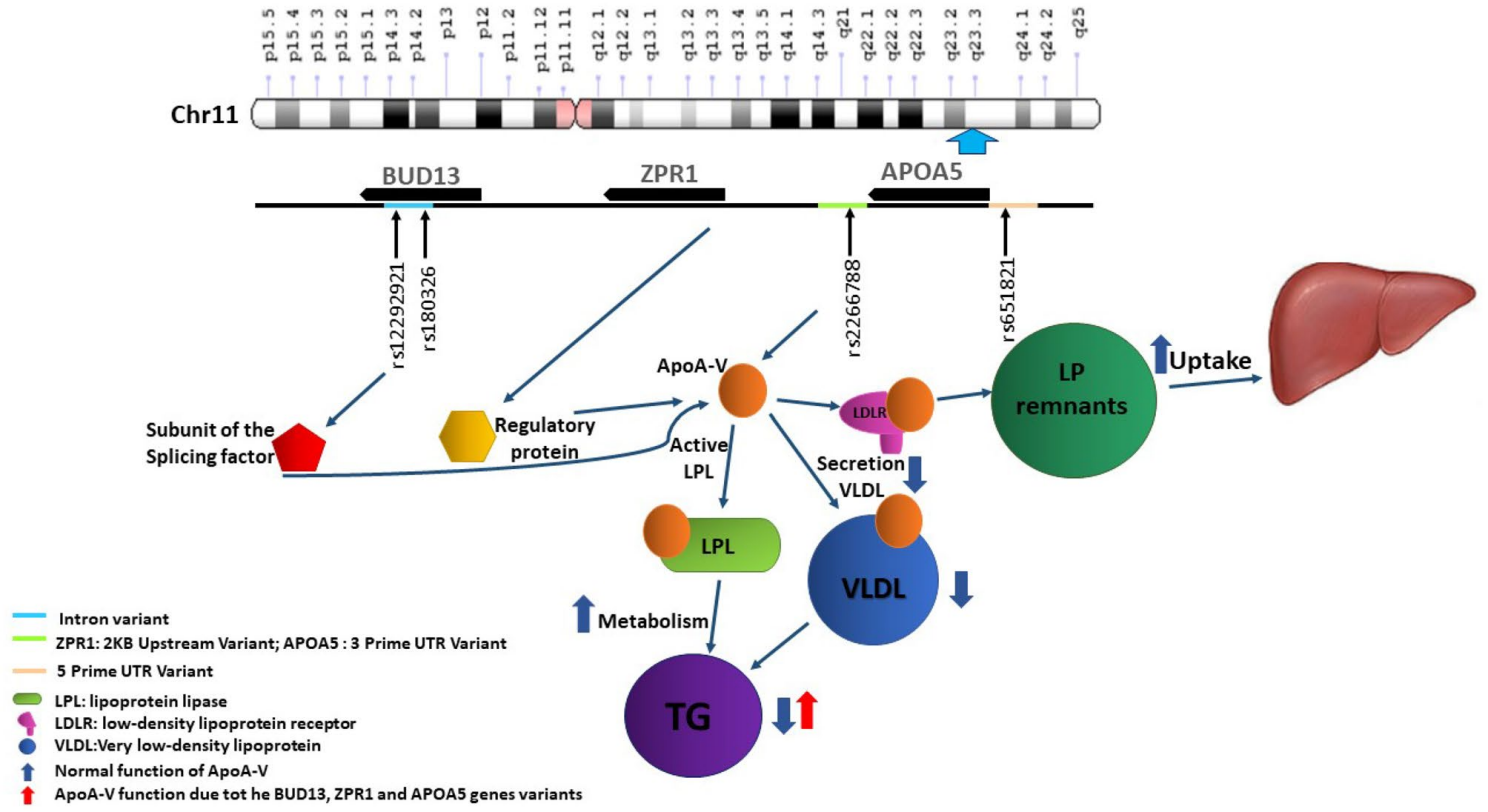
Schematic picture for the BUD13, ZPR1, and APOA5 genes on the 11p23.3 region, location of four notable significant SNPs, and shows the mechanism of ApoA-V action on triglyceride level.

The *BUD13, ZPR1*, and *APOA5* genes encode three proteins in the *APOA5* protein pathway in the 11q23.3 chromosomal region. *BUD13* is a subunit of the splicing factor that plays a role in preserving nuclear pre-mRNA, and *ZPR1* is a regulatory protein related to signal transduction and cell proliferation^27^. *APOA5* protein plays an important role in several mechanisms, including insulin sensitivity, glucose, fatty acid, and cholesterol metabolism^15,16^. The *APOA5* protein is an LPL activator, which inhibits hepatic VLDL production, and accelerates the hepatic uptake of lipoprotein remnants and insulin secretion. Furthermore, it is related to free fatty acid decrease, triglyceride synthesis, and stimulates adipose tissue^25,28^. It is claimed that interaction between *BUD13/ZPR1* variants and *APOA5* may change its function and increase the TG level^7,29,30^ (Fig. 4).

Many studies in different populations, including European and Asian populations, confirmed that *BUD13, ZNF259*, and *APOA5* variants correlate with changes in plasma TG levels^11,31,32^. Studies on SNPs in noncoding, exonic, and intergenic regions showed transcriptional binding sites of adjacent genes lead to transcriptional processes affected by the SNPs without being linked explicitly to protein regulation^27^.

The GWAS study conducted on the Chinese population indicated that rs651821 on *APOA5* and rs180326 on *BUD13* are associated with MetS and serum TG levels^7^. Another GWAS study showed the homozygotes carriers of rs2266788-G *APOA5* has pleiotropic effects on (increasing) TG and (lowering) HDL-C levels^13^. Also, as a functional point mutation, rs2266788 is highly correlated with rs651821^7^.

Our results are consistent with Korean and Kuwaiti population studies^25,26^, which reported rs2266788 as related variant hypertriglyceridemia (TG > 150 mg/dL) with similar OR. Our results are also consistent with a study performed by Karja et al. to confirm the rs2266788 risk allele concerning increased TG^13^. The results of a linkage disequilibrium replicated by Zhu et al. indicated a strong LD between rs2266788 and rs651821. Furthermore, the association of rs2266788, rs651821, rs180326, and rs12292921 with MetS and HTg-MetS were reported in our outcome. Although the first three SNPs' association in our study and the global studies were deep-rooted, rs12292921 is not well known^7,33–36^.

Finally, the present study was done in well-characterized and extended TCGS participants who were monitored for more than twenty years. The kernel machine SNP set analysis by two-step models was used for the first time to evaluate this genetic relationship; this would be the most important strength of these findings. The limitation of this research includes the failure to check the APO cluster associated with the lipid profile.

In conclusion, the kernel machine and Single SNP Regression models found the association of genetic variations on the *BUD13, ZPR1*, and *APOA5* genes (11q23.3 region) with MetS and High TG levels in MetS affected individuals in the TCGS population. We suggested using these methods in the same studies. Moreover, rs2266788 and rs651821 in, *APOA5* gene was in strong LD and showed a significant association with MetS and HTg-MetS and can be used for prediction. These two SNPs also showed the most significant association with HTg-MetS, which indicates that this region is related to changing lipid profiles in metabolic syndrome. Therefore, we recommend a more in-depth study in the APO genes cluster to confirm these findings.

## Materials and methods

### Subjects and population

Subjects selected from Tehran Cardio-metabolic Genetic Study (TCGS)^37^. This study is an ongoing genetic cohort study designed to determine the risk factors of major non-communicable disorders in the Tehran Lipid and Glucose Study (TLGS)^38^, whose subjects were followed-up cardio-metabolic risk factors over twenty years. The attendance enrolled in the study from 1999 until now, and over twenty thousand participants were monitored for seven phases (1999–until now; periodic follow-up interval: 3 years). An expert obtained informed consent from each subject and then interviewed for collecting demographic data and referred each one to trained physicians and laboratories for clinical examinations and blood sampling at each visit; details are published elsewhere^37,39,40^. All procedures performed in this study approved by the ethics committee on human subject research at Research Institute for Endocrine Sciences, Shahid Beheshti University of Medical Sciences (code of “IR.SBMU.ENDOCRINE.REC.1395.366”), which were following the 1964 Helsinki Declaration and its later amendments or comparable ethical standards.

In the present study, 5421 unrelated individuals more than 18 years old were recruited. According to the Iranian modified metabolic syndrome joint interim statement (JIS) definition, the metabolic syndrome is defined for all participants^41,42^ who attended the sixth phase of TCGS (2015–2017).

We defined two models to assign individuals to the case and control groups. In the first model (Mets versus non-MetS), the cases were selected as MetS affected if they had at least three of the following criteria^41^: (1) abdominal obesity (increased WC ≥ 91 cm in females and males) based on national cut-offs^42^, (2) TG ≥ 150 mg/dl or receiving treatment for hypertriglyceridemia, (3) HDL < 50/40 mg/dl in F/M, (4) SBP ≥ 130 mmHg or DBP ≥ 85 mmHg or receiving treatment for hypertension, (5) FPG ≥ 100 mg/dl or previously diagnosed with type 2 diabetes and diabetic drug users. The rest of the population were selected as controls^42^.

**Figure 5:**
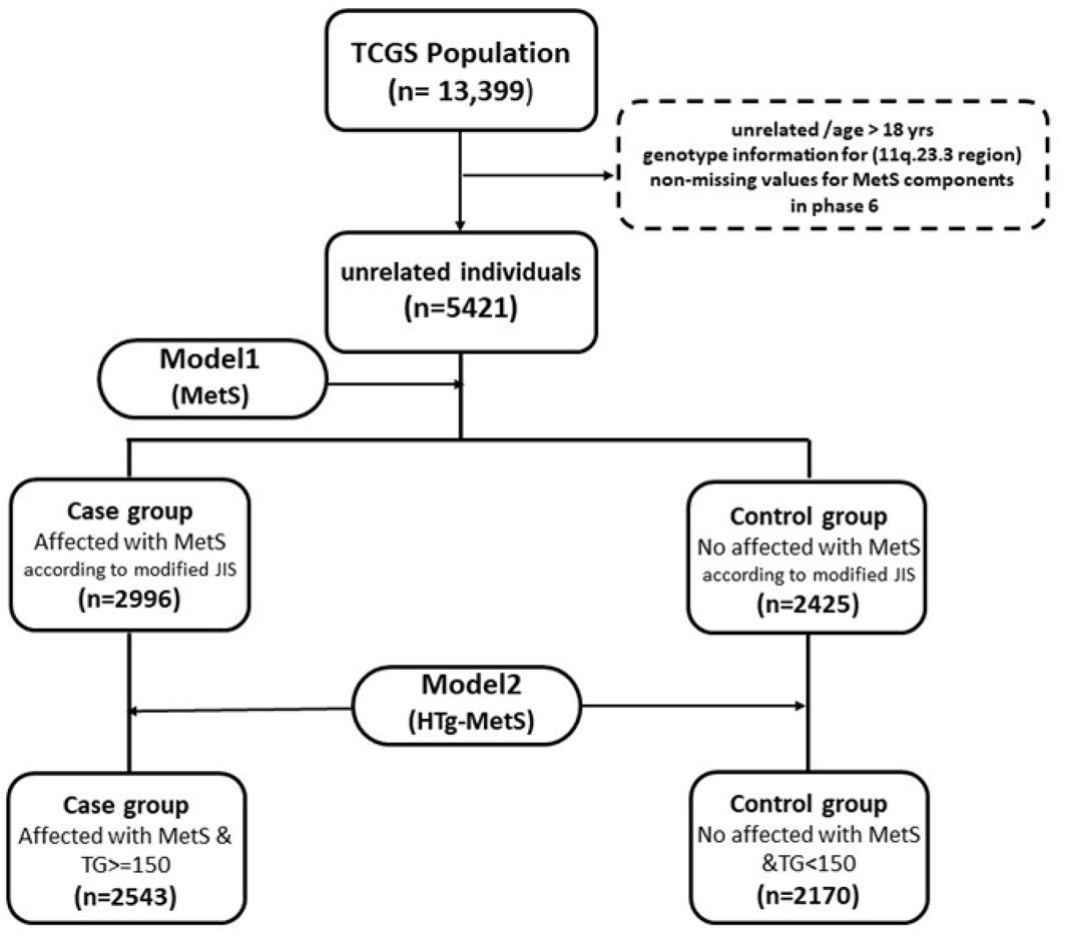
Flow diagram of the study. Model 1 and Model 2 shows by MetS and HTg-MetS symbols, respectively.

For the second model to measure precise triglyceride effect on association analysis (hTG-MetS), subjects were divided into case and control if they affected by MetS at the same time have TG higher than 150 mg/dl and Non-MetS with TG less than 150 mg/dl, respectively, and other individuals were excluded (Fig. 5).

### Genotyping and SNP selection

DNA samples were extracted from white blood cells by a standard Proteinase K, salting-out method. The accuracy and quality control^43^ of extracted samples were evaluated with electrophoresis and spectrophotometry. DNA samples of TCGS participants were genotyped with HumanOmniExpress-24-v1-0 bead chips at the deCODE genetics company (Iceland) according to the manufacturer's specifications (Illumina Inc., San Diego, CA, USA)^37^. After quality control for markers and individuals in our desired region, 20 SNPs located on the BUD13, ZPR1, and APOA5 genes were selected. According to our defined inclusion criteria and genotyping After omitting 2 SNPs with departure from Hardy–Weinberg equilibrium, 18 SNP remained in our analysis^44^. (Exact test p < 0.01) (Table 2). The plink software was used to create a list of minor allele frequencies (MAF) for SNPs^45^, and the 1000 Genomes database was used to find global minor allele frequencies for comparison^46^.

### Statistical analysis

#### Descriptive

All continuous variables were expressed as mean and standard deviation, whereas categorical variables are summarized as frequencies and percentages. Differences between the two groups were calculated using the chi-square test or t-student test. The significance of deviations of observed genotype frequencies from those predicted by the Hardy–Weinberg equation evaluates the χ^2^ test with statistical significance at the level of 0.01. Rare variants, SNPs with minor allele frequency (MAF) less than 0.01, were not considered for further analysis.

#### Haploview

In Haploview software version 4.2, four gamete reules procedure was used to make SNP sets from correlated adjacent SNPs in our region^47^. For each marker pair, the four possible two-marker haplotypes’ population frequencies are computed; if all four were observed with at least a frequency of 0.01, it is deemed to recombination have occurred. According to this analysis, SNPs were clustered into three sets. According to linkage disequilibrium (LD), the LD plot and haplotypes were drawn under the four gamete rule, with a connection between genotyped SNPs and the tested regions of the *BUD13, ZPR1*, and *APOA5* genes in the 11q23.3 chromosomal region^47,48^.

#### Kernel machine regression

To increase the association test's power and accommodate the epistasis effect between adjacent correlated SNPs in a set, we used a logistic kernel machine regression method to assess SNP sets' association binary phenotype^23,28,49,50^. In the additive model, genotypes AA, Aa, and aa recoded to 0, 1, and 2, respectively^28,50^.

#### Single SNP analysis

We considered an additive logistic regression model to assess each SNP's association in the significance sets with binary phenotype. We used the PLINK2 program and P values based on MAX (T) procedure and permutation test on the additive effect (with 1 million iterations)^45,51^. The risk allele for each associated marker was labeled, and the presence of the risk allele was classified into three genotype groups (Homozygote for reference allele (HomoRef = 0), Heterozygote = 1, Homozygote for the alternate allele (HomoAlt = 2)) in all significant SNPs. For the four most significant SNPs genotypes, MetS components level were compared by one-way ANOVA and Tukey test (Table 5). The triglyceride level was normalized, and the logarithm (log) of TG for each individual was plotted against genotypes. Then, LogTG was compared in different genotypes by ANOVA and Tukey test to find significant genotype groups (Fig. 3). Finally, We used the PLINK2 program to calculate Pairwise r2 and D′ values between rs2266788 and rs651821.

### Ethics approval

All procedures followed the ethics committee's ethical standards on human subject research at Research Institute for Endocrine Sciences, Shahid Beheshti University of Medical Sciences.

### Consent to participate

Informed consent was obtained from all the participants.

### Consent for publication

All co-authors and responsible authorities have approved this study's publication at the institute.

## Data Availability

In this study, the information of participants was from TLGS and TCGS studies.
